# Detection of *Salmonella* spp. from chevon, mutton and its environment in retail meat shops in Anand city (Gujarat), India

**DOI:** 10.14202/vetworld.2015.388-392

**Published:** 2015-03-23

**Authors:** P. P. Makwana, J. B. Nayak, M. N. Brahmbhatt, J. H. Chaudhary

**Affiliations:** Department of Veterinary Public Health and Epidemiology, Anand Veterinary College, Anand Agricultural University, Anand, Gujarat, India

**Keywords:** food safety, meat, prevalence, *Salmonella* spp, serotype

## Abstract

**Aim:**

The aim of this study was (i) To attempt isolation and identification of *Salmonella* species from samples. (ii) Serotyping of *Salmonella* isolates. (iii) Detection of virulence factor associated genes by polymerase chain reaction (PCR).

**Materials and Methods:**

A total of 284 samples comprised of chevon and mutton (112 samples each) as well as 60 samples (20 each of retail meat shops environment samples *viz*. Butchers’ hands, knives and log swabs) were collected from the retail meat shops in and around Anand City under aseptic precautions. Rappaport-vassiliadis soy bean meal broth and tetrathionate broth was used for the enrichment of all the samples and inoculation was done on brilliant green agar and xylose lysine deoxycholate agar. This was followed by the confirmation of isolates using biochemical tests. For the serotyping, isolates were sent to the National Salmonella and Escherichia Centre, Central Research Institute, Kasauli, Himachal Pradesh. Detection of virulence genes was performed by PCR technique using previously reported primer.

**Result:**

Of 284 meats and retail meat shops environment samples, 13 (4.58%) samples were found positive for *Salmonella*. It was interesting to know that incidence of *Salmonella* was more in mutton (6.25%) than chevon (3.57%). In case of meat shop environmental samples 1 (5.00%) sample observed positive for *Salmonella* separately among the butchers’ hands and knives swabs (Each of 20 samples) examined. Out of 13, eleven isolates detected as *Salmonella* Typhimurium, whereas only two isolates were detected as *Salmonella* Enteritidis. All *Salmonella* isolates possess *inv*A and *stn* genes, whereas nine isolates had a presence of *spv*R gene while only five of the isolates revealed the presence of *spv*C gene as shown by *in vitro* detection of virulence genes by PCR.

**Conclusion:**

Therefore, might be suggested that the good hygiene practices and effective control measures should be taken to encourage clean meat production with prolonged shelf-life.

## Introduction

Population growth has increased the requirements for an expanded food industry production [1]. In these industry production, *Salmonella* remains in first place of world’s leading causes of bacterial food borne illness [2]. The first outbreak of salmonellosis reported during the late 1800’s in which 57 people affected that ate beef. Due to *Salmonella* infections 93.8 million cases of gastroenteritis reported in year worldwide, with 155,000 deaths. Milder infections of *Salmonella* are mostly under-diagnosed; therefore the actual cases of infections may be very high [3]. The financial losses occurred due to *Salmonella* infections have drawn increasing attention in developed countries in recent years.

Animals are exposed to *Salmonella* in many ways (i.e. water, feed, feces, soil, and insects) and can become infected or asymptomatic carriers of the *Salmonella* organism [3]. People become infected with *Salmonella* by contaminated food and water. *Salmonella* infection primarily spread from contaminated areas by human and Animals activities to other animals and areas.

Chevon and mutton are valuable source of protein and it is frequently consumed by many communities in India, specifically at religious event celebration. Goats and sheep are mostly slaughtered at small abattoirs having not so much hygienic conditions in most parts of India [4]. The poor hygienic conditions in the slaughterhouses and meat shops encourage microbial contamination, survival and growth [5].

Thus, the aim of this study was to detection of *Salmonella* spp. from chevon, mutton and its environment in retail meat shops.

## Materials and Methods

### Ethical approval

All the procedures have been carried out in accordance with the guidelines laid down by the Institutional Ethics Committee and in accordance with local laws and regulations.

### Sample collection

From July 2013 to March 2014, a total of 284 samples comprised of chevon and mutton (112 samples each) were excised with a sterile scalpel under aseptic conditions into sterilized polythene bags from the retail meat shops in and around Anand city. Each bag was labeled indicating code number and other particulars of the sample. Moreover, 60 samples (20 each of retail meat shops environment samples *viz*. Butchers’ hands, knives and log swabs) were taken into cairy blair transport medium and placed in thermocol box containing ice and brought to the departmental post-graduate laboratory for further processing and microbiological analysis.

### Isolation and identification

Samples were processed as per standard protocol described in bacteriological analytical manual (BAM), U.S. Food and Drug Administration (USFDA) method [6] with necessary modifications.

**Figure-1:**
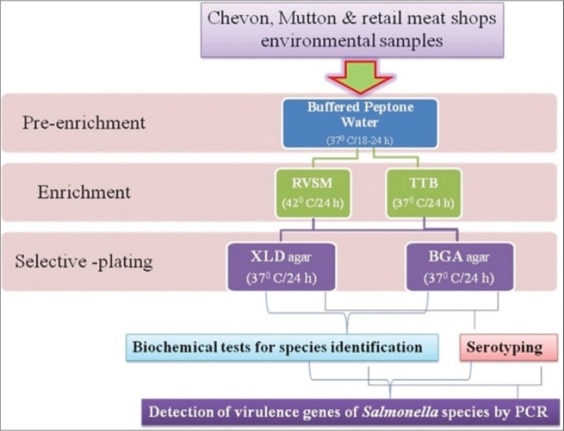
Procedure for isolation of *Salmonella* from chevon, mutton and retail meat shops environmental samples.
b) Retail meat shop environment samples: Swab collected from butchers’ hands, knives and log will be directly inoculated in pre-enrichment broth and further processed in a similar manner as mentioned above.

a) Chevon and mutton: 25 g of the meat sample will be homogenized with 225 ml of lactose broth in a stomacher blender and pre-enriched at 37°C for 24 h., approximately 0.1 and 1 ml of pre-enriched samples are transferred to rappaport-vassiliadis soy bean meal broth and tetrathionate broth, respectively, followed by 24 h of incubation at 42 and 37°C, respectively. The enrichments are streaked on brilliant green agar and xylose lysine deoxycholate agar and incubated for 24 h at 35°C. All presumptive *Salmonella* colonies will be inoculated on triple sugar iron (TSI) agar and incubated at 37°C for 24 h and further biochemical characterization of the isolates will be carried out Figure-1.

### Biochemical examination

Biochemical tests were performed to confirm *Salmonella* Spp. Using catalase test, oxidase test, indole test, citrate test, urease test, voges proskaur (VP) test and H_2_S production TSI (Table-1).

**Table-1:** Biochemical characteristics of *Salmonella.*

Test	Reaction
Catalase	+
Oxidase	-
H_2_S production (TSI)	+
Indole test	-
MR test	+
VP test	-
Citrate test	+
Urease test	-

TSI=Triple sugar iron, MR=methyl red, VP=voges proskaur

Serotyping of *Salmonella* isolates: *Salmonella* isolated from samples were serotyped at the National Salmonella and Escherichia Centre, Central Research Institute, Kasauli, H. P., India.

DNA isolation: Extraction of DNA from *Salmonella* was done by using boiling method [7]. Approximately a loopful of culture was taken in a micro centrifuge tube and mixed with 100 µl of sterilized DNAse and RNAse free water. This was followed by denaturation at 95°C for 10 min using the thermal cycler (Applied Biosystems, Sweden). Finally, cell debris was removed by centrifugation (10000 rpm for 5 min) and 3 µl of the supernatant was used in the PCR as DNA-template.

### Detection of virulence genes by polymerase chain reaction

All the *Salmonella* isolates were first screened for the presence or absence of virulence associated genes by using the PCR protocols separately standardized for the detection of different genes. The PCR was standardized for the detection of four genes *viz. inv*A, *spv*R, *spv*C and *stn* following the methodology as described by [8-11] respectively, with suitable modifications. Standardization of PCR was done by using standard strain of *Salmonella* Typhimurium (VP81) (Table-2).

**Table-2:** Primer pairs used for virulence associated genes characterization of *Salmonella* isolates.

Target genes	Primer sequence (5’→3’)	Product size (bp)	Reference accession no	References
*inv*A	F: GTG AAA TTA TCG CCA CGT TCG GGC AA	284	AE 006468.1	7
	R: TCA TCG CAC CGT CAA AGG AAC C			
*spv*R	F: CAG GTT CCT TCA GTA TCG CA	310	AE 006471.1	8
	R: TTT GGC CGG AAA TGG TCA GT			
*spv*C	F: ACT CCT TGC ACA ACC AAA TGC GGA	571	AE 006471.1	9
	R: TGT CTT CTG CAT TTC GCC ACC ATC A			
*Stn*	F: CTT TGG TCG TAA AAT AAG GCG	260	AE 006468.1	10
	R: TGC CCA AAG CAG AGA GAT TC			

F=Forward primer, R=Reverse primer

## Results and Discussion

### Prevalence of *Salmonella* spp.

In the present study, it was revealed that, out of 284 samples comprised of chevon and mutton (112 samples each) as well as 60 samples (20 each of retail meat shops environment samples *viz*. Butchers’ hands, knives and log swabs), 13 (4.58%) samples were found positive for *Salmonella* spp. (Table-3) according to cultural characteristics and biochemical tests (Table-1). The occurrence of *Salmonella* was more in mutton (6.25%) than chevon (3.57%). Finding of the present study was in concordance with Kumar *et al*. [12] and very low than the previously reported more than 5.0% prevalence [13].

**Table-3:** Source wise prevalence of *Salmonella* spp.

Source of samples	Meat samples		Environmental swab samples			Total no of positive sample
	
	Chevon	Mutton	Butchers’ hands	Knives	Log	
Shop 1	1 (3.57)	1 (3.57)	1 (20.00)	ND	ND	3 (4.22)
Shop 2	1 (3.57)	3 (10.71)	ND	ND	ND	4 (5.63)
Shop 3	2 (7.14)	1 (3.57)	ND	1 (20.00)	ND	4 (5.63)
Shop 4	ND	2 (7.14)	ND	ND	ND	2 (2.82)
Total	4 (3.57)	7 (6.25)	1 (5.00)	1 (5.00)	ND	13 (4.58)

Note: Figures in parenthesis indicate percentage, ND=Not detected

### Serotyping of *Salmonella* isolates

There were eleven isolates detected as *S*. Typhimurium, while two isolates were detected as *Salmonella* Enteritidis. Serotype of individual isolate is shown in Table-4. This result correlates well with Selvaraj *et al*. [14] who reported *S*. Typhimurium and *S*. Enteritidis as the predominant serovars in India from different animal sources. Whereas other authors reported various other serotypes like *Salmonella* Infantis Yadav *et al*. [15], *Salmonella* Saintpaul and *Salmonella* Chester [16], *Salmonella* Kissi [17].

**Table-4:** Isolate wise serotype and prevalence of virulence genes.

Isolate no	Serotype	Virulence gene			

		*inv*A	*spv*R	*spv*C	*Stn*
C1-09	Typhimurium	+	+	+	+
C2-17	Typhimurium	+	-	+	+
C3-25	Typhimurium	+	+	-	+
C3-02	Typhimurium	+	+	-	+
M1-05	Typhimurium	+	+	-	+
M2-03	Typhimurium	+	+	-	+
M207	Enteritidis	+	-	-	+
M2-23	Typhimurium	+	+	-	+
M3-11	Typhimurium	+	-	-	+
M4-08	Typhimurium	+	+	+	+
M4-27	Enteritidis	+	+	-	+
BH1-05	Typhimurium	+	-	+	+
K3-02	Typhimurium	+	+	-	+

### Detection of virulence gene by PCR

**Figure-2:**
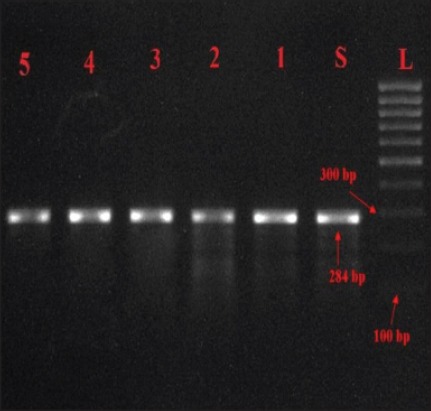
Agarose gel showing PCR amplified products (284bp) for *inv*A gene in *Salmonella* isolates

**Figure-3:**
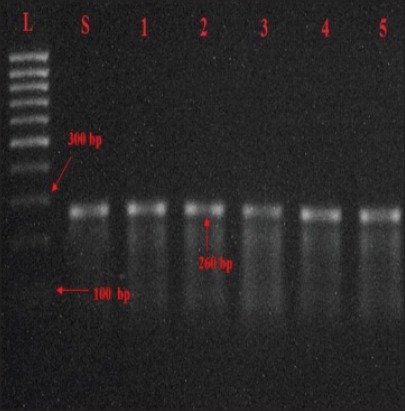
Agarose gel showing PCR amplified products (260bp) for *stn* gene in *Salmonella* isolates

Out of 13 *Salmonella* isolates all yielded desired amplified products of 284 bp and 260 bp for *inv*A and *stn* genes, respectively as shown in Figures-2 and 3 similar results have also been reported by Mir *et al*. [18] and Singh *et al*. [19].

**Figure-4:**
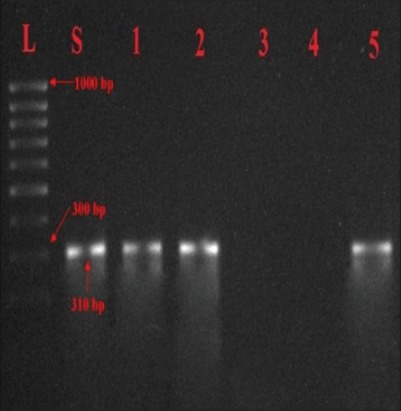
Agarose gel showing PCR amplified products (310bp) for *spv*R gene in *Salmonella* isolates

Whereas, nine of the isolates produced 310 bp product specific for *spv*R (Figure-4). In contrast to our results Oliveira *et al*. [10] found that 91.20% *Salmonella* Enteritidis isolates contained the *spv*R gene. Bessa *et al*. [20] reported that out of 66 *Salmonella* Typhimurium strains 4.54 % positive for *spv*R by PCR assay.

**Figure-5:**
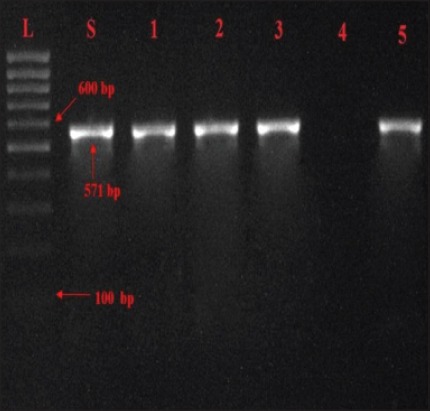
Agarose gel showing PCR amplified products (571bp) for *spv*C gene in *Salmonella* isolates

While only five isolates of *Salmonella* yielded 571 bp product specific for *spv*C gene (Figure-5) which correlate well with Das *et al*. [21] who reported that 42.85% isolates yielded *spv*C gene from 35 *S. enterica* isolates by PCR assay.

## Conclusion

Different serotypes isolated from these environmental sources, majority of these serotypes were of zoonotic significance and thus, these places require appropriate hygiene to avoid cross contamination of the meat. A thorough sanitation procedure not only prevents potential hazard to human health but also creates the clean surroundings.

Data profiles of this study also use for establish direction and help to evaluate control strategy of meatborne disease related to *Salmonella* bacteria.

## Authors’ Contributions

PPM supervised the overall research work. PPM and JHC participated in analysis of samples and made available relevant literatures. JBN and MNB participated in draft and revision of the manuscript. All authors read and approved the final manuscript.
